# Address on Tobacco

**Published:** 1847-10

**Authors:** A. Berry


*Address on Tobacco, delivered before the Mississippi Valley Association of Dental Surgeons, at its Third Annual Meeting, by* A. Berry, D. D. S.

Mr. President and Gentlemen:

Having been appointed at our last annual meeting to deliver an address on the use of Tobacco, at this anniversary, I proceed to discharge the duty, fearful, however, that I shall fail to interest you in what I may offer.

Tobacco was discovered by the Spaniards when they invaded South America, and by them introduced into Spain and Portugal. It was taken to England by Sir Frances Drake, in 1586, and to France about the same time, by John Nicot, as a curiosity.

The use of this plant, perhaps the most disgusting to the natural appetite of any substance in the vegetable kingdom, soon spread throughout the civilized world. It met with considerable opposition from intelligent, and philanthropic men; but this seemed only to make the evil more extensive. The civil and ecclesiastical authorities interposed and attempted to eradicate it ere age had given it vigor.

James I, noted for his learning, good sense, and zeal for the welfare of his subjects, in his Counterblaste to Tobacco, denounces it as "sinful and shameless lust," and warns them "not to sin against God and harm their own goods, and render themselves scorned and contemned by strangers who should come among them, by persevering in a custom loathsome to the eye, hateful to the nose, baneful to the brain, dangerous to the lungs, and in the black and stink, fume thereof, nearest resembling the horrible Stygian smoke of the pit that is bottomless." He also prohibited any Virginia planter from raisng more than one hundred pounds of Tobacco annually.

Pope Urban VIII, published a decree of excommunication against all who should be guilty of taking snuff in the church.

In Russia, smoking tobacco was prohibited under penalty of having the nose cut off.

Tobacco was brought to Constantinople by the Dutch, in 1617, and its use was at first strongly opposed by the Mufti, as a violation of the Koran. A Turk found smoking, was led through the streets of the city with his pipe transfixed in his nose. But the Grand Vizier becoming fond of the weed, distributed it in rations to the Janizaries. This powerful body soon silenced all opposition; and thenceforth the Moslemuna sat in security beneath the Crescent, although enveloped in clouds from their favorite pipes.

The Puritan Fathers of New England, distinguished above all other people for purity of morals, and integrity of purpose, endeavored by severe enactments, to prevent the use of tobacco in their commonwealths.

All efforts to prevent the use of this weed, proved futile. In every country where it can be obtained, the high and the low, the rich and the poor, the learned and the ignorant, the moral and the vicious, the priest ministering at the altars of our holy religion and the conjuror of most debasing heathen rites, all luxuriate in the powers of this triumphant plant. It is regarded as a potent auxiliary to human happiness. In the hour of suffering and adversity, it assuages pain and dispels the weighty sorrows of the mind; and in the enjoyment of every luxury, this seems to afford the greatest delight.

Tobacco possesses properties narcotic, sedative, diuretic, emetic, cathartic, and errbine. In moderate doses it produces a languor very grateful to those accustomed to it. In large doses it causes vertigo, nausea and general debility of the nervous and circulating functions, which may terminate fatally. It yields by distillation, ompyreumatic oil, which is a virulent poison. A case is recorded of a child, whose death was caused by rubbing oil from the bowl of a pipe on a ringworm. A similar result followed the application of expressed juice of tobacco leaves to the head of a child for tinea capitis.

Mr. Brodie applied one drop of the oil to the tongue of a cat, and in fifteen minutes, when it had apparently recovered from its effects, he applied another drop and produced death in five minutes.

Dr. Mussey, in a considerable number of experiments, found that three drops of the oil rubbed on the tongue of a full sized cat, caused death in from three to ten minntes, and in one instance, in two minutes and forty-five seconds.

Does the use of tobacco injure the health? The habitual use of tobaoco weakens the functions of the digestive apparatus, and deranges the nervous system, and must, in this way, be indirectly prejudicial to the teeth. Sound and healthy teeth and gums in the mouth of a person of delicate health, are an anomaly.

Most dental writers, who have mentioned the subject, consider tobacco injurious to the teeth.

Fouchard says, "the smoking tobacco is also very injurious to the teeth; it makes them black and ugly; and besides, if the precaution is not taken to cover the end of the pipe, the rubbing which it makes against the teeth never fails, in the using of it, to uncover their sensible part."

Langbotham remarks, "the smoking or chewing this herb is frequently introduced for the vehement pain of the tooth ache, and with most constitutions, paves the way to a far more dangerous disease than it is intended to remove, by its acrid and internally violent qualities; and the chemical oil which it leaves within the hollow of the teeth, disposes them to blackness and premature decay, which, though less obnoxious for the present, proves a lasting enemy to the mouth and stomach.

Dr. Fitch says: "there is an impurity or acrimony about some tobacco, which causes it to injure the teeth; but my observations as to the effects of good tobacco are, that by some individuals it may be chewed with impunity, and that smoking tobacco may be allowed, and probably used as an errbine, it never injures the teeth; but as it is a dirty, and in many instances, pernicious substance, perhaps its use might, with advantage, be entirely dispensed with."

Dr. Mussey, whose long and extensive medical practice and habits of close observation entitle his remarks to great weight, says: "the opinion that the use of tobacco preserves the teeth is supported neither by physiology or observation. Constantly applied to the interior of the mouth, whether in the form of cud or smoke, this narcotic must tend to enfeeble the gums and the membrane covering the necks and roots of the teeth, and in this way must rather eccelerate than retard their decay. We accordingly find, that tobacco consumers are not favored with better teeth than others; and on the average, they exhibit these organs in a less perfect state of preservation. Sailors make free use of tobacco, and they have bad teeth."

On the leaves of tobacco when gathered, there is always some dust from the soil that is not entirely removed in the process of manufacturing, which, by its mechanical action, abrades the teeth in chewing the cud. And if the dropping of water wears away stones, will not the constant grinding of tobacco, under the pressure of the masseter muscle, wear away the teeth?

I have seen several cases of tobacco chewers who, before they had lived half of the three score and ten, had worn the teeth on which they masticated their cud, so as to make the corresponding sides of their faces shorter than the other sides. They assured me that they could not quit the use of tobacco, and that to chew it on the teeth unaccustomed to it, produced nausea. In one of these cases considerable deformity was caused by the wearing away of the front teeth of one side; while those of the other remained of full length.

The narcotic and nauseating properties of tobacco, may remove the pain of an aching tooth, for which it is frequently prescribed, instead of the forceps. A gentleman of my acquaintauce, being directed by his physician to chew tobacco for the toothache, continued the practice for thirty years, when upon mature deliberation he concluded he had chewed long enough for a pain that never occurred but once, and sagely resolved to abandon the filthy habit. He did so, but not without experiencing considarable suffering for several days.

The opinion that chewing tobacco preserves the teeth from decay is, I think, eroneous. It arises, probably, from the fact that many persons find their teeth to decay less rapidly after commenceing its use than before; but they resort to it at a time when their teeth contain a larger proportion of lime than at an earlier period, and are therfore less liable to be affected with caries. And besides, people generally take better care of their teeth, after they arrive at the age in which they usually commence chewing tobacco than previously to that time, preventing in a great degree the causes of their decay.

The pressure of the cud on the gums of the molares and hicuspides, not unfrequently causes them to recede, leaving the roots of these teeth exposed and liable to decay; while the gums are in many cases inflamed, and seldom in a perfectly healthy condition.

The smoking of tobacco injures the teeth by the sudden changes of temperature, to which it exposes them. It renders the mouth most disgustingly filthy, coating the inner surfaces of the teeth with black empyreumatic oil, exhaling an odor like that of a much used tobacco pipe, and inducing diseased action in the gums.

In some sections of our country rubbing snuff on the teeth and gums, called dipping, is in common and popular u[illegible] especially among the ladies It is applied with little swabs or brushes made by chewing the ends of pieces of soft wood. The pretended reason for dipping—and no one could indulge in so filthy and disgusting a habit without some excuse for it—is that it preserves the teeth from decay. A "social dip" requires some half a dozen participants, each with a brush, which they all dip into one bottle of snuff and rub their teeth and gums for a considerable length of time; while the stimulus of the snuff adds excitement to the conversation. By this practice the snuff is frequently impacted in the interstices of the teeth, and insinuated beneath the edges of the gums, which is sometimes so extensive in its effects as to cause a loosening of the teeth. From several years observation of the teeth of a large number of persons who use snuff in this manner, I am induced to believe, that it has no tendency to preserve the teeth from decay; and that they are more likely to be attacked with caries from the general diseased state of the gums.

In many instances the snuff communicates to the teeth a dirty yellowish tinge, very unpleasent in its appearance. To no young lady long accustomed to dipping, would the language of an Indian to his lady-love be appropriate: "Mila has the eyes of the ermine; and the hair of a field of rice; *her mouth is a rosy shell garnished with pearls.*"

It may be proper for us as servitors of the health of the teeth, to inquire what we can do to remove the evils resulting from the use of tobacco. To this question, I answer, we should exhibit a good example in reference to it. Tobacco should be put under the same ban with alcoholic drinks. We should touch not, taste not the unclean thing. We should on all proper occasions, give our testimony against indulging in the filthy, health destroying habit of using tobacco in any form.

